# Relations between Nonsuicidal Self-Injury and Suicidal Behavior in Adolescence: A Systematic Review

**DOI:** 10.1371/journal.pone.0153760

**Published:** 2016-04-18

**Authors:** Salome Grandclerc, Diane De Labrouhe, Michel Spodenkiewicz, Jonathan Lachal, Marie-Rose Moro

**Affiliations:** 1 Maison de Solenn, Hôpital Cochin, AP-HP, Paris, France; 2 Université Paris Descartes, Sorbonne Paris Cité, Paris, France; 3 Université Paris-Saclay, Univ. Paris-Sud, UVSQ, CESP, INSERM, Villejuif, France; 4 Service de Psychiatrie de l’Enfant et de l’Adolescent, Groupe Hospitalier Pitié-Salpêtrière, APHP, Paris, France; 5 Université Pierre et Marie Curie, Paris, France; 6 Institut de Systèmes Intelligents et Robotique, Paris, France; University of Western Brittany, FRANCE

## Abstract

Nonsuicidal self-injury (NSSI) and suicidal behaviors, both important issues in adolescent health care, are frequently associated and possibly clinically related. Our objective was to explore the views of relations between nonsuicidal self-injury and suicidal behaviors during adolescence and young adulthood (11–25 years) expressed in the scientific (medical and psychological) literature. We adopted a textual approach to the process of synthesis to tell the story of the findings from the included studies. Our narrative systematic review of 64 articles found that they share the same risk factors. Integrated models envision nonsuicidal self-injury as a gateway enabling teens to acquire the capability for suicide. Because suicidal behavior short-circuits thought, it is difficult to conceive an intention to die during adolescents' acts of self-injury. Intention is constructed by the narrative of the act, influenced by numerous elements from the psychopathologic, cultural, religious, and philosophic context. Techniques of mentalizing-based treatments and work on the meaning that adolescents attribute to their behaviors might improve care.

## Introduction

Nonsuicidal self-injurious behavior (NSSI), also referred to as self-mutilation (for example, cutting, burning or hitting oneself, scratching oneself to the point of bleeding and interfering with healing) is a relatively frequent behavior in adolescents and young adults (jointly described hereafter as young people [1]) and is reported to affect around 10% of them [2–6]. Its principal risks are that it will become chronic and evolve toward other forms of self-injurious behavior, such as suicide attempts [4]. The frequent observation of the coexistence of NSSI and suicidal behavior [7,8] requires that we consider the nature of the link between these two types of behavior as well as the ways that NSSI is conceptualized. Its relation to suicide is equivocal: some specialists envision NSSI behavior as a means of maintaining life by reducing and regulating negative emotions [9,10], while others argue that it is a factor precipitating the emergence of suicidal ideation and attempts [10,11].

In the United States, the mean prevalence of NSSI in the clinical population of hospitalized adolescents is around 35% [4,12–14], while its prevalence in the general population of teens is thought to be around 10% [2–6]. Some of these adolescents present recurrent NSSI behavior [8,15]. Many authors identify adolescence as a period at risk for NSSI behavior: it begins most often during puberty, at the age of 13 to 15 years [4,16,17], and its prevalence falls in adulthood [18]. Girls begin this type of behavior earlier than boys [19] and are therefore at higher risk during adolescence [2,20]. This gender difference nonetheless tends to attenuate with age [21,22]. Finally, studies show that the incidence of NSSI in western countries is highest among white male adolescents [18,23].

Suicide is also an important concern in adolescent health: it is the second leading cause of death among those aged 15–29 years [24], and 4 to 8% of American adolescents present suicidal behavior between the ages of 15 and 19 years [13,18,25]. The prevalence of suicidal behavior is still higher among adolescents hospitalized in psychiatric wards [14,26]. Suicidal behaviors appears to progress over time: suicidal ideation is more common at the beginning of adolescence [13,25,27], the incidence of suicide attempts highest at the end of adolescence [13,25,28], and the number of suicide deaths increases as adulthood begins [13,25,27]. Although suicide attempts are more frequent among young girls than boys, a gender difference in the suicide mortality rate is less clear in this age group than among adults [2,25,27,29].

The risk of both suicide attempts and suicide is significantly higher in those who have engaged in NSSI. Among those with a history of NSSI, 70% have attempted suicide at least once and 55% several times [8,30]. The risk of death by suicide is highest during the first 6 months after an NSSI episode and tends to fall later on [31]. Adolescence and young adulthood is a period at risk for both types of behavior [18,30].

NSSI and suicidal behavior also raise problems in terms of their management: access to care is often difficult for adolescents (50% do not seek help at all, 30% contact social workers, and only 20% ask for medical treatment) [32], and this care is heterogeneous, often performed by people who are barely trained and often believe many of the stereotypes and old wives' tales about NSSI [4,33,34].

The distinction between NSSI and suicidal behavior is not always clear in the terminology used–a lack of clarity that illustrates their conceptual proximity [35]: numerous English terms cover NSSI [36], and the choice of term depends on the theoretical and clinical environment. Three expressions are used most often: *self-injury*, *deliberate self-harm* and *nonsuicidal self-injury* (NSSI). One such term is *self-injury* which includes NSSI but also behavior such as exposing oneself to violent situations, trichotillomania, ingesting drugs, etc. Another is *deliberate self-harm* which comprises all NSSI behavior and intentional drug overdoses. The term we use here, *nonsuicidal self-injury* (NSSI) [37], was chosen to designate intentional and non-socially acceptable behaviors that are intended to cause destruction or impairment of bodily tissues but only minor or moderate physical harm, performed without any conscious suicidal intention, self-directed, and used to reduce psychological distress [38–40]. Despite the numerous variations in the definitions of these clinical populations, their prevalence rates remain remarkably stable [41]. In view of the multiplicity of terms, some authors have attempted to structure a concept of NSSI and to distinguish it from suicidal behavior. Karl Menninger's classification [42] organizes self-injurious behaviors in five categories: destruction, suicide, chronic suicide, focal suicide, and organic suicide. Favazza [43], on the other hand, classes self-mutilations into three phenomenological categories: major (amputations), stereotypical (found in autism), and finally moderate superficial, the most common among adolescents (NSSI). The latter is subdivided into compulsive (trichotillomania, onychophagy), often repetitive, ritualized, and occurring several times a day, and impulsive (cutting or burning), which may be episodic or repeated. The switch from impulsive episodic to repetitive NSSI generally occurs after an average of 5 to 10 episodes. Walsh's classification [9] resembles that of Favazza: it differentiates the types of NSSI according to the degree of lethality of the act, the manner of its performance (direct or indirect), and whether it occurred once or repeatedly.

These and other attempts at definition and classification share a concern for clarity and the particularity of being simultaneously descriptive and atheoretical [35]. The existing classifications were elaborated with the aim of coming as close as possible to the concept of NSSI, studying its association with psychiatric diseases, and individualizing among the NSSI one or more subtypes that might correspond to syndromes. Nonetheless, the combination of characteristics of these behaviors produces a constellation of profiles of self-harming individuals; this multiplicity explains the difficulty in evaluating and managing them. A better understanding of this phenomenon and an attempt to identify an NSSI group at high risk of suicide would make it possible to develop more effective evaluation tools. Searching for NSSI in patients with suicidal behavior and vice versa should become systematic [44]. Pattison and Kahan [45] have suggested work in this direction: they sought to include in DSM-III a deliberate self-harm syndrome, in the category of impulse control disorders. Favazza [43] had a similar aim in describing a "syndrome of repetitive impulse dyscontrol with protean symptoms". More recently, NSSI behaviors were a topic of discussion in the development of DSM 5 [46]. Because the data were inadequate for any conclusion, NSSI appears instead in the section "Conditions for Further Study", as a distinct syndrome.

### Objectives

In our daily clinical practice, NSSI and suicidal behaviors appear to be closely linked. We thus decided to survey what the literature says about their associations and to examine how research and theory envision them. Accordingly, the objective of our narrative systematic review is to examine the association between NSSI and suicide in young people (age 11–25 years) in the scientific (medical and psychological) literature, focusing on papers that study the intentions underlying these acts. Specifically, we seek to refine the nosographic classification of these phenomena. The issue is to pinpoint these behaviors and their reciprocal interactions to improve their assessment and their management and thus to limit their often serious complications. We present the different integrated models that have attempted to establish an association between NSSI and suicide and discuss their limitations. This requires a better characterization of NSSI, which can be simultaneously seen as a symptom of various psychiatric disorders, an individualized syndrome, or part of a spectrum of self-injurious behavior that includes suicide. We have chosen to apply a textual approach to the process of synthesis in order to tell the story of the findings from the studies included here.

## Method

This is thus a narrative systematic review of the topic of the associations between suicidal and NSSI behaviors in adolescents. Narrative systematic review makes it possible to synthesize findings from multiple studies when statistical meta-analysis is not feasible [47] (S1 PRISMA).

This inductive systematic work includes six steps:

1. Definition of the research question and the objectives
2. Manual literature review to select the principal papers on the topic and to construct key words and inclusion and exclusion criteria
3. Systematic review and identification and selection of studies
4. Analyzing the papers, extracting their data and identifying their themes
5. Generating a thematic analysis and structuring the synthesis
6. Writing the paper

Our clinical experience with adolescents presenting self-injurious behaviors has led us to question the heterogeneity of their profiles and the apparent associations between NSSI and suicide (step 1). An initial literature search [35,48] allowed us to refine this question to focus on the intentions underlying these acts. First, we identified publications by the principal international authors who have examined this research question; we included 24 articles. Next, we constructed a list of key words from these articles and determined the inclusion and exclusion criteria (step 2). Then, we conducted a systematic web search on Medline and PsycInfo with the following key words "NSSI" [OR] "deliberate self-harm" [AND] "suicidal behavior" [AND] "adolescence". Papers were selected only if they met the following criteria:

- Published in English
- Between January 1990 and January 2014
- Considered the association between NSSI and suicide
- Adolescent and young adulthood population (11 to 25 years when specified)
- All methodologies were included (quantitative as well as qualitative or mixed studies).

Finally, the following studies were excluded:

- Studies focusing on specific medical conditions such as psychosis, autism, mental disabilities, or chronic somatic suffering (diabetes or chronic pain for example)

**Fig 1:**
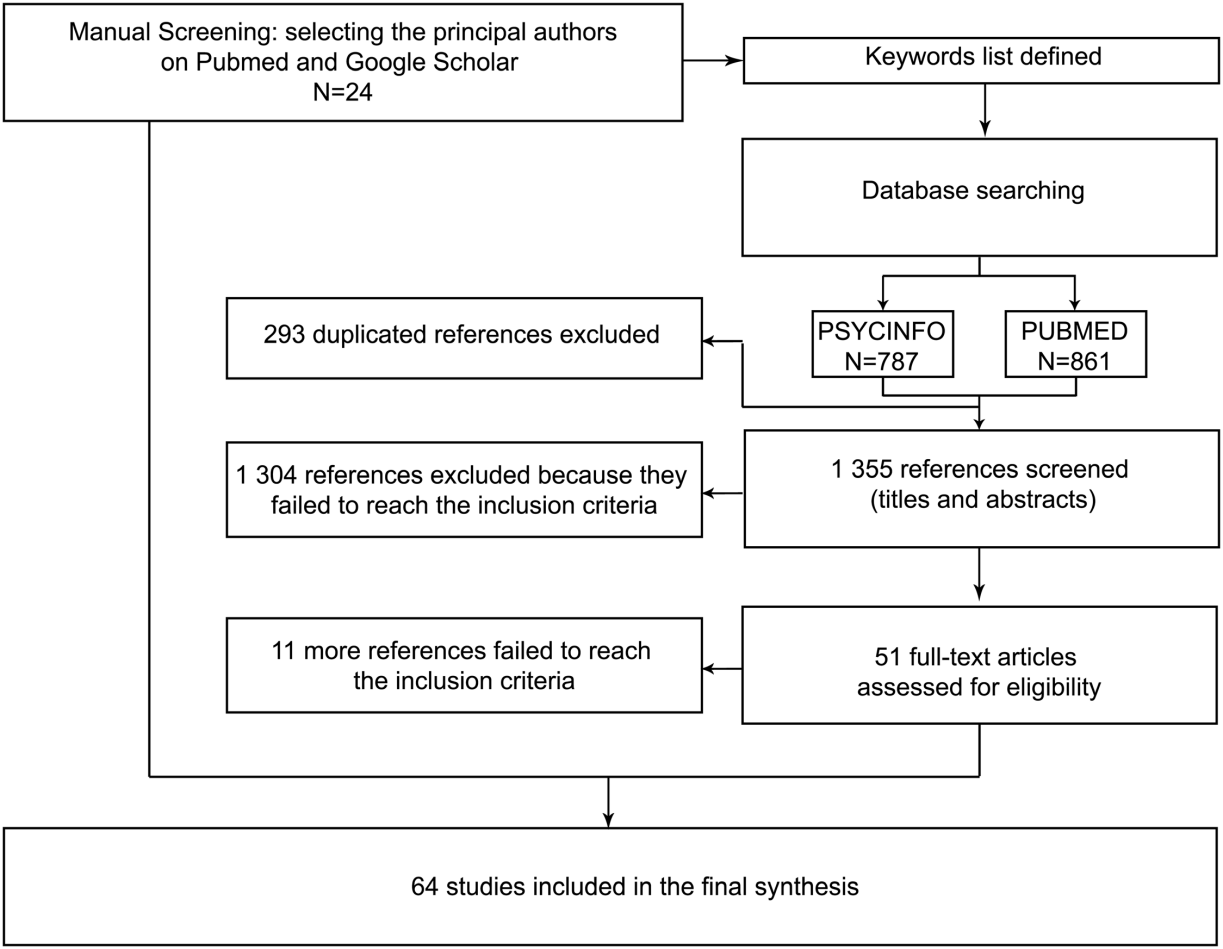
Flowchart. Fig 1 summarizes the selection of the articles included in this literature review.

After elimination of duplicates, we obtained 1355 references (Fig 1). Two authors (SG and DDL) screened all titles and abstracts, according to their relevance. This first selection allowed us to exclude 1304 references that did not meet the inclusion criteria. After reading the full-text of the remaining 51 articles, we included 40 articles in addition to the initial 24. Thus, the review finally included 64 studies (step 3). The detail of our protocol is provided in S1 File, and the characteristics of the studies are provided in S1 Table. Fig 1 presents the flow chart (S2 PRISMA).

The data extraction was performed after reading carefully the complete articles. Three researchers (SG, DDL and JL) independently extracted and analyzed the data and developed categories inductively from the themes identified in these studies (step 4). The data were then reported descriptively, cross-classified, shared, and distributed throughout the various subthemes, in a narrative synthesis. As our objective was to explore the views of the relations between NSSI and suicidal behaviors in literature, we have chosen to focus on words and narrative results instead of statistical results per se. Results were nonetheless extracted according to their level of relevance and regrouped according to their common themes. Finally we ordered these results into a framework containing two themes (step 5). The last step consisted in expressing the synthesis in a useful form (step 6). This process led not to a summary of the different studies included, but to an interpretation of the papers, described in the discussion.

This review consists of a thematic construction in an analytic framework of data from this research, intended to present a narrative report of the existing literature on the nosographic questions concerning self-injurious behaviors.

## Results

In all, we examined 64 studies that questioned relations between NSSI and suicidal behaviors in adolescence and young adulthood. S2 Table details the characteristics of each study. The thematic analysis of scientific literature clearly showed two themes. The first is an exploration of the association between suicidal behaviors and NSSI and comprises four subthemes: *shared risk factors*, *the contribution of intergroup comparisons*, *whether NSSI is a risk factor for suicide or not*, and *intentions underlying NSSI and suicidal behaviors*. The integrated models are the second theme and include three subthemes: *the gateway theory*, *the third variable theory*’ and *Joiner's theory*: *pain tolerance and capability for suicide*.

### Exploration of the Association between Suicidal Behaviors and NSSI

The association between NSSI and suicidal behavior is expressed in different ways. Here we describe some different aspects of this relation, organized in four subthemes.

#### The shared risk factors

First, NSSI and suicidal behavior share some risk factors [8,49]:

- among the psychiatric comorbidities: depression, borderline personality disorder [8,20], substance abuse, posttraumatic stress disorder, impulsivity, externalizing behaviors [8,30,50], attention deficits, with or without hyperactivity, and conduct disorders [4].
- among the contextual, relational and traumatic factors: history of sexual abuse or physical violence [51], family dysfunction [1,12,52,53].

#### The contribution of intergroup comparisons

Some authors have suggested the potential value of intergroup comparisons [3,7,13,14,18,19,21,26,54–56]: adolescents or young adults with NSSI alone (NSSI group), compared to those with NSSI and suicidal behavior (NSSI+SB group), or those with suicidal behavior alone (SB group).

Young people with both types of behavior (NSSI+SB) are at higher risk of psychiatric symptoms or at least of psychological vulnerability than the other two groups (NSSI group and SB group). For example, the adolescents in the NSSI+SB group are diagnosed more frequently than the others with major depressive episodes or posttraumatic stress. They more often present symptoms of self-denigration, anhedonia, and impulsivity. They also report more suicidal ideation and fewer reasons to live. This suggests that the multiplicity of means of self-injury forms a group of patients with more clinically severe disease [54]. Moreover, family dysfunction is more common in this NSSI+SB group, although problems with their friends and peers are not. Comparing the young people with a history of NSSI alone (no history of suicide attempts) to those with past suicide attempts but no NSSI, we note that the former have fewer psychiatric symptoms (depressive and post-traumatic symptoms). The group with NSSI alone has the fewest psychosocial dysfunctions.

#### Is NSSI a risk factor for suicide or not?

From another perspective, numerous works have studied the correlations between NSSI and suicidal behaviors. Some authors treat NSSI as an important risk factor predictive of concomitant or subsequent suicidal behavior or ideation [26,28,31,44,57,58]. Recent studies have made it possible to rank risk factors for suicide according to their level of correlation with attempted suicide. Most highly correlated are frequent NSSI and use of several different NSSI methods, in second and third position, behind suicidal ideation and ahead of some psychiatric symptoms (borderline personality disorder, impulsivity, posttraumatic or depressive symptoms) and demographic characteristics (gender, ethnicity, or age) [2,33,59,60].

Nonetheless, there are numerous methods of self-injury [43], and some appear to be at higher risk than others of leading to suicidal behavior. NSSI behaviors with the following characteristics are associated with significantly higher rates of suicidal acts: duration longer than one year, higher number of methods used [19,61], cutting, high frequency of NSSI, absence of physical pain during the act, severe physical damage, strong conscious intention to die, and concealment of the action [8]. These characteristics must be assessed for an accurate evaluation of NSSI severity.

Other authors argue that NSSI should be envisioned as protective against suicidal behavior. This is the anti-suicide model [62]. NSSI is considered a compromise to avoid total destruction by channeling destructive impulses into a circumscribed area [42,63]. NSSI could thus serve as acts of microsuicide that create an illusion of control of death [63]. This model stresses its coping/self-regulation function, aimed at preventing suicide. Some adolescents thus say that they have used NSSI to reduce the suicidal ideation against which they struggle [58].

#### Intentions underlying NSSI and suicidal behaviors

What elements distinguish NSSI from suicidal behavior? The central difference is that suicide attempts involve a conscious intention to die, through the abolition of consciousness [35,64]. Accordingly, while the suicidal act is seeking death, the objective of NSSI injury seems to be to relieve unbearable emotions, by seeking to modify rather than abolish the state of consciousness. Recourse to multiple methods of self-injury with a low risk of death and a high frequency of acts (the frequency of NSSI is substantially higher than that of suicide attempts; teens can average 20 to 30 NSSI acts a year) underlines the function that NSSI behaviors serve: emotional self-regulation and relief from psychological pain [35]. The reasons adolescents give to justify their self-injurious behavior can be classified as intrapersonal—to seek relief from negative effects or on the contrary to seek feelings to reduce their experience of anhedonia—or interpersonal—to communicate their malaise, ask for help, or escape from a difficult situation [10,17,32,34,65–68]. The recent definition of NSSI insists on the absence of a conscious intention to die and distinguishes—by opposing—NSSI and suicide attempts [46].

Nonetheless, intentionality appears to be a theoretical difference, difficult to clarify clinically: it complicates the question. It is difficult for mental health professionals to assess clinically what adolescents think and say about death, especially their (the adolescents') own, for these thoughts and accounts depend on their character, education, and culture. Young people most often mix together their seeking of relief and their ideas of death [67,69]. Differentiating NSSI from suicidal behavior in clinical practice on the basis of intentionality is therefore complex. These behaviors appear to have multifactorial determinants [66] that might be more easily accessible by considering a continuum of self-injurious behavior that includes both NSSI and suicidal behavior.

### Integrated Models

Seeking a more thorough theoretical comprehension of this topic, several authors have set themselves the task of understanding why and how NSSI is a predictive factor for suicidal behavior for some patients but not for others. Integrated models have proposed several readings of the link between NSSI and suicide.

#### The Gateway theory

Some began by envisioning the concept of a continuum or spectrum [3,70,71], considering NSSI and fatal suicides as two ends of the same spectrum, two different manifestations of the same behavior. NSSI may represent an antechamber to suicide; it is this alarm value that requires particular attention [35]. This analysis is based on one of the first models for understanding the association between NSSI and suicide: the gateway theory [3,13,70]. As seen above, NSSI is a highly predictive risk factor for suicidal behavior [26,28,54,57,72]. This theory is supported by both retrospective and prospective studies, after adjustment for other risk factors [13,31,44,54]. NSSI might therefore be a single and independent risk factor for subsequent suicidal behavior. Several points support this hypothesis: the strong co-occurrence of these two types of behaviors underlies their association [14,26,73,74]; NSSI also begins early, according to the epidemiologic data [13,18,23,25,29,41] and thus appears to precede suicidal behavior. NSSI triples the risk of subsequent but also concomitant suicidal behavior [44]. It is nonetheless thought of as a one-way predictive factor, with suicide attempts not considered to predict risk of NSSI [13,26,58]. NSSI is accordingly considered a gateway toward more severe forms of self-injury.

#### The Third Variable theory

A second model assumes the existence of a third variable, the presence of which links NSSI and suicidal behavior (the Third Variable theory). It is based on the sharing of risk factors and on the high prevalence of similar psychiatric diseases in young people who have died by suicide and those with NSSI (90% vs 87%) [13,75]. The variables to be taken into account might include a depressive state, suicidal ideation, personality disorder, low self-esteem, or unsupportive family [3,15,16]. For example, the presence of borderline personality disorder simultaneously increases the risk of NSSI and of suicidal behavior. Thus consideration of this variable would make it possible to use NSSI to predict suicide attempts [14]. NSSI also appear to be strongly predictive of suicidal behavior among a population of depressed adolescents [76]. The identification of a group of NSSI patients at risk of suicide would therefore depend on the presence of a third variable.

#### Joiner's theory: Pain tolerance and capability for suicide

The two models presented above envision the association between NSSI and suicidal behavior restrictively, with NSSI considered either as a risk factor (Gateway Theory), or as a comorbidity (Third Variable Theory). They have major theoretical and clinical limitations and have been undermined by some recent studies [13,26,54]; new models have therefore become necessary.

Towards this end, Joiner [11] developed an integrated model of self-injurious behaviors and was able to develop different explanations for why individuals whose life course had until then been marked by NSSI would attempt suicide. It is based on an original approach: pain tolerance. Other authors have since supported and added to this model [77–79].

Joiner [11] conserved the concept of a continuum ranging from NSSI to suicidal behavior, but added a variable coming from neurosciences—modulation of pain [80], which is driven by the endogenous opioid and endocannabinoid pathways [81]. The repetition of NSSI might accordingly disrupt the pathways involved in the stress-induced analgesia that leads to the phenomenon of pain tolerance [82]. The author thus suggests that when a person cannot imagine or represent his or her own death, NSSI appears to be an acceptable alternative. NSSI could thus be considered a way of acquiring a capability for suicide. A strong association has been found between the use of a high number of NSSI methods, a high frequency of NSSI, and the risk of suicide attempts by self-injuring adolescents [60]. NSSI might thus enable the person to become accustomed and thus desensitized to the fear and pain of physically hurting oneself [11,13]. The more varied the means of NSSI and the more frequent its episodes, the greater the increase in capability for self-injury and then for suicide [59].

Reinforcing this theory, Muehlenkamp and Gutierrez [18] show that self-injuring adolescents describe less fear of suicidal acts than those without this NSSI history. The self-injuring adolescents also appear to show greater pain tolerance during standardized tasks than control subjects [13,83,84]. We can thus ask whether NSSI is a means of pain desensitization [85] or if these self-injurers have a greater constitutional tolerance than others [83]. Finally, the association between NSSI and the acquisition of capability for suicide may be associated with the severity of nonsuicidal self-injurious behavior. Subjects with severe forms of NSSI may be at higher risk of acquiring this capability [13].

Several authors see NSSI as a strategy of emotional adaptation and regulation [59,65]. If this strategy fails, the adolescent must undertake more severe forms of self-injury, which become progressively closer to suicidal behavior [15]. Nonetheless, here, NSSI is considered as one of many behaviors that can contribute to the acquisition of this capability and can accordingly increase the risk of suicide. Among them, drug or alcohol abuse and exposure to violence, such as combat experience, can also favor a gradient in self-injury [13,78]. Joiner's model [11] thus has a much wider theoretical reach: it makes it possible to analyze the relation of numerous behaviors and impulsive actions.

The variable of capability for suicide has direct clinical interest for both primary and secondary prevention. Identifying a subtype of patients at higher risk would make it possible to provide graduated management and more supportive care to the patients at the greatest risk of suicidal acts [60].

## Discussion

Our literature review has allowed us to explore diverse aspects of the relation between NSSI and suicidal behavior. All of these behaviors directed against the self share the same terrain of fragility and risk factors; they are also statistically correlated. NSSI thus appears to be a risk factor predictive of subsequent suicidal behavior. This result has been used in the development of several integrated models, the most recent of which includes the concept of acquiring the capability for suicide through NSSI. Most of the studies on this subject begin by distinguishing NSSI and suicidal behaviors according to the intention to die. This intention appears to us to be central but also very difficult to assess in clinical situations. The definition of behaviors with and without death as their intention has made it possible to set up groups of patients accessible to research. Nonetheless, the assessment of adolescents with such different profiles and clinical histories seems close to impossible to us. In our view, this question cannot be resolved completely, both because adolescents cannot imagine death at the moment they are acting out these behaviors and because of the act’s communicative value. NSSI and suicide attempts thus appear to be behaviors on a single continuum of self-injury. We think that a distinction based on the intentionality of the action does not justify the conclusion that a desire for death was not present during NSSI nor does it differentiate NSSI from suicidal behavior.

The question is what a desire to die can mean to young people. Behind intentionality lies the question of the representation of one's own death at this age. Adolescence is not only a period of construction and transition, but also the stage when awareness of death develops. Adolescents are confronted with the need to grieve for the immortality of their childhood. The concept of death appears to follow a developmental progression [86], as found in children. Representations of childhood overlap with adult representations, those transmitted by environment, culture, and religion.

It is thus both frequent and normal for adolescents to think about death. Adolescents regularly develop interests in symbols of death and in music groups that convey these symbols. These thoughts can be considered to be a necessary psychological development: with puberty, the feeling of death, anxiety about death, and ideas of death, seen as irreversible, universal and inevitable, fuse to approach knowledge of death [87–89]. The objective is to understand, become familiar with, and ascertain the limits of life and death.

Nonetheless, one's own death is unthinkable: according to some authors, death is an ontological impasse [90]. It makes no sense, has no meaning in itself, and may not be entirely accessible to human reason. The issue of adolescence is thus to construct a symbolic representation of death in order to control it or at least to defuse its threat [87,91]. Adolescents when they are performing a self-directed act, are no longer in a symbolic elaboration but in an action that expresses their inability to imagine death [87]. The act short-circuits the thought. There would thus be neither a desire nor an absence of desire to die during the act.

Seeking to assess the intentionality underlying the act thus appears contradictory. In NSSI as in suicide attempts, the act short-circuits the thought. Before the act, death is thought, symbolized: it may appear fascinating, attractive, or not. After the act, it becomes urgent to create a narrative of the event. This is the moment when intentionality appears to be constructed, in an attempt to give a meaning to the act. At this stage, adolescents may or may not allow themselves to talk about suicidal behavior.

Numerous elements can interfere with this process (of meanings). In the first place, the reaction of family and friends and the relational value they give to the act appear central. We know the communicative value given to self-injury, which is sometimes interpreted as aggression or rejection [1,92]. But the cultural environment must also be taken into account, and the taboo of suicide, whether legal, moral or religious, can influence this psychological work [93–96]. Finally, the influence of psychopathologic processes, such as depression, cognitive disorders, and addictions, plays a role [8,13,76]. All of these dimensions—psychopathologic, cultural, religious, and philosophic—are necessary to understand self-injurious acts.

Additional studies thus appear necessary to examine this question in greater detail, to take into account the complexity of the context, and to explore further the subjective experience reported by patients. Qualitative studies are perfectly adapted for the in-depth study and detailed understanding of these complex questions of behaviors [97].

### Implications for practice

It is possible to associate the difficulties presented by adolescents who self-harm with defective mentalization [98]. Mentalization is understood here as the capacity to understand oneself and to understand others by deducing the mental states that underlie their apparent behavior: their thoughts, beliefs, intentions, motivations, and goals. This fundamental psychological process is at the interface of numerous mental disorders, so that this theory may find a generic application in psychiatric care [99–101]. In the case of the adolescents we study here, their inability to represent death may thus be accessible to the methods of treatment proposed, for example, for borderline personality disorders (mentalization-based treatment) [98]. The principal work of the therapist in this treatment is to encourage their patients' curiosity, their continual questioning of their own mental state, of the way they do things. The therapist's task is to aid adolescents in understanding how to find a meaning in their experience. We propose to apply this treatment technique to the management of adolescents who self-harm, to promote their capacity to represent NSSI behavior or suicidal acts and the emotional states associated with them, and accordingly to modify the meaning of these acts.

## Supporting Information

S1 File Description of the method. (DOC)

S1 PRISMA PRISMA Checklist. (DOC)

S2 PRISMA PRISMA Flow Diagram. (DOC)

S1 Table Characteristics of studies. (DOCX)
