# Supplementary material for: Relations between Nonsuicidal Self-Injury and Suicidal Behavior in Adolescence: A Systematic Review
Source: PLoS One. 2016 Apr 18;11(4):e0153760. doi: 10.1371/journal.pone.0153760 (PMC4835048; doi:10.1371/journal.pone.0153760)
Supplement: S1 File — (DOC) [file pone.0153760.s001.doc]

**S1 File – Description of the method**

1. **Definition of the objectives**

- Studying the links between NSSI and Suicidal attempts
- Focus on the intentions underlying these acts
- In adolescence and young adulthood (11 – 25 years)

1. **Manual literature review**

- Inclusion of 2 books (Fox & Hawton, 2004; Gicquel & Corcos, 2011)
- Screening the references and selecting the principal authors on the topic
  - MS Andover
  - L Claes
  - K Hawton
  - TE Joiner
  - JJ Muehlenkamp
  - MK. Nock
- Screening the publications of these authors on pubmed and google scholar
  - Inclusion of 24 papers

1. **Systematic review**

- Search terms specified in research meetings with
  - Keywords from the first included papers
  - Search terms used in other works (Lachal, Orri, Sibeoni, Moro, & Revah-Levy, 2015; Orri et al., 2014)
- Inclusion and exclusion criteria specified in research meetings

1. Inclusion criteria

- Published in English
- Between January 1990 and January 2014
- Considered the association between NSSI and suicide
- Adolescent and young adulthood population (11 to 25 years when specified)
- All methodologies were included (quantitative as well as qualitative or mixed studies)

1. Exclusion criteria

- Studies focusing on specific medical conditions such as psychosis, autism, mental disabilities or chronic somatic suffering (diabetes or chronic pain for example)
- Complete search strategy. Databases from 01/1990 to 01/2014

PUBMED (Filters: English, 01/01/1990 to 01/31/2014)

((MH “Suicide+”) OR (MH “Suicidal Ideation”) OR (MH “Suicide, Attempted”) OR (“suicide Attempts”) OR (“suicide”) OR (“attempted suicide”) OR (“suicidal ideation”) OR (“suicide ideation”) OR (“suicidal behaviour”) OR (“youth suicide”)) AND ((MH “Self mutilation”) OR (MH “Self-Injurious Behavior+”) OR (“self inflict*”) OR (“self harm*”) OR (“self cut*”) OR (“self destruct*”) OR (“self mutilate*”)) AND ((MH “Adolescent”) OR (MH “Adolescent Psychology”) OR (MH “Adolescent Psychiatry”) OR (MH “Adolescent Behavior”) OR (MH “Adolescent Development”) OR (“teenagers”) OR (“teens”) OR (“adolescence”) OR (“adolescent”) OR (“adolescents”) OR (“young”))

n= 861

PSYCINFO (Filters: English, Peer reviewed journals, 01/1990 to 02/2014)

((DE “Suicide+”) OR (DE “Attempted Suicide”) OR (DE “Suicidal Ideation”) OR (“suicide Attempts”) OR (“suicide”) OR (“attempted suicide”) OR (“suicidal ideation”) OR (“suicide ideation”) OR (“suicidal behaviour”) OR (“youth suicide”))

AND ((DE “Self mutilation”) OR (DE “Self Injurious Behavior”) OR (DE “Self Destructive Behavior”) OR (“self inflict*”) OR (“self harm*”) OR (“self cut*”) OR (“self-injur*”) OR (“self mutilate*”))

AND ((DE “Adolescent Psychiatry”) OR (DE “Adolescent Psychology”) OR (DE “Adolescent Psychopathology”) OR (DE “Adolescent Psychotherapy+”) OR (DE “Adolescent Attitudes”) OR (DE “Adolescent Development”) OR (“teenagers”) OR (“teens”) OR (“adolescence”) OR (“adolescent”) OR (“adolescents”) OR (“young”))

n=787
