# Supplementary material for: Relations between Nonsuicidal Self-Injury and Suicidal Behavior in Adolescence: A Systematic Review
Source: PLoS One. 2016 Apr 18;11(4):e0153760. doi: 10.1371/journal.pone.0153760 (PMC4835048; doi:10.1371/journal.pone.0153760)
Supplement: S2 PRISMA — (DOC) [file pone.0153760.s003.doc]

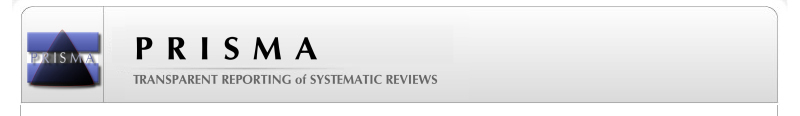
**PRISMA 2009 Flow Diagram**

**Screening**

**Included**

**Eligibility**

**Identification**

Records identified through database searching
(n = 1648 )

Additional records identified through other sources
(n = 24 )

Records after duplicates removed
(n = 1379 )

Records screened
(n = 1379 )

Records excluded
(n = 1304 )

Full-text articles assessed for eligibility
(n = 75 )

Full-text articles excluded, with reasons
(n = 11 )

Studies included in qualitative synthesis
(n = 64 )

Studies included in quantitative synthesis (meta-analysis)
(n = N/A )
