# Supplementary material for: Relations between Nonsuicidal Self-Injury and Suicidal Behavior in Adolescence: A Systematic Review
Source: PLoS One. 2016 Apr 18;11(4):e0153760. doi: 10.1371/journal.pone.0153760 (PMC4835048; doi:10.1371/journal.pone.0153760)
Supplement: S1 Table — (DOCX) [file pone.0153760.s004.docx]

| **References** | **Design** | **Country** | **Sampling Design** | **Aims** | **Use of Validated Instruments** | **Results & conclusions** |
| --- | --- | --- | --- | --- | --- | --- |
| Andover, M. S., & Gibb, B.E. (2010).  Non-suicidal self-injury, attempted suicide, and suicidal intent among psychiatric inpatients. *Psychiatry Research*, *178*(1), 101‑105 | Original retrospective study | USA | 117 psychiatric inpatients in a general hospital (adults and adolescents). | Examining the relations between NSSI and attempted suicide among psychiatric inpatients with self-report questionnaires. | Self-report questionnaires:  -Suicidal Behaviors Questionnaire (SBQ)  -Suicide Intent Questionnaire (SIQ). | -Presence and number of NSSI episodes were significantly related to presence and number of suicide attempts.  -Patients' history of NSSI (presence and frequency) was strongly associated with history of suicide attempts. |
| Andover, M. S., Morris, B. W., Wren, A., & Bruzzese, M. E. (2012).  The co-occurrence of non-suicidal self-injury and attempted suicide among adolescents: distinguishing risk factors and psychosocial correlates.  *Child Adolesc Psychiatry Ment Health*, *6*, 11‑11 | Review | USA | N/A | To investigate the association between attempted suicide and NSSI among adolescents. | N/A | -Attempted suicide associated with poor family functioning, suicidal ideation, unstable self-concept, and conduct problems, and NSSI associated with hopelessness, presence of an anxiety disorder, female gender, and younger age.  - NSSI is a significant predictor of subsequent NSSI and subsequent suicide attempts. |
| Andover, M. S., Primack, J. M., Gibb, B. E., & Pepper, C. M. (2010).  An Examination of Non-Suicidal Self-Injury in Men: Do Men Differ From Women in Basic NSSI Characteristics?  *Archives of Suicide Research*, *14*(1), 79‑88 | Original retrospective study | USA | 48 individuals reporting a history of NSSI (adolescents and young adults) among a nonclinical sample. | Describing and comparing basic NSSI characteristics among a nonclinical sample by gender. | Interviews:  -Frequency of Activities Scale  -Checklist-90-Revised  -Self-Mutilative Behaviors Interviews  -Self-Injury Scale  -Global Severity Index. | Men and women differed significantly on age of onset, degree of medical injury, and NSSI methods. |
| Anestis, M. D., Bagge, C. L., Tull, M. T., & Joiner, T. E. (2011).  Clarifying the role of emotion dysregulation in the interpersonal-psychological theory of suicidal behavior in an undergraduate sample. *Journal of Psychiatric Research*, *45*(5), 603‑611 | Original retrospective study | USA | 283 undergraduates. | To examine two specific components of emotion dysregulation – negative urgency and distress tolerance – and their relationships to all three components of the interpersonal-psychological theory of suicidal behavior. | Self-report questionnaires:  -Urgency Premeditation Perseverance Sensation seeking and Positive Urgency Impulsive Behavior Scale (UPPS-P)  -Distress Tolerance Scale (DTS), Acquired Capability for Suicide Scale (ACSS)  -Interpersonal Needs Questionnaire (INQ). | -Emotionally dysregulated individuals - with low distress tolerance and high negative urgency - exhibited higher levels of suicidal desire, as indexed by perceived burdensomeness and thwarted belongingness.  -Emotionally dysregulated individuals exhibited lower levels of the acquired capability for suicide and physiological pain tolerance |
| Asarnow, J. R., Porta, G., Spirito, A., and al. (2011). Suicide Attempts and Nonsuicidal Self-Injury in the Treatment of Resistant Depression in Adolescents: Findings from the TORDIA Trial.  *J Am Acad Child Adolesc Psychiatry, 50*(8), 772‑781 | Original prospective randomized study | USA | 334 depressed adolescents who did not improve with an adequate SSRI trial randomized to a medication switch (SSRI or venlafaxine), with or without cognitive-behavioral  therapy. | To evaluate the clinical and prognostic significance of suicide attempts (SAs) and nonsuicidal self-injury (NSSI) in adolescents with treatment-resistant depression. | Questionnaires:  -Schedule for Affective Disorders and Schizophrenia for School-Aged Children (K-SADS)  -Clinician Weekly Rating Scale  -Brief Suicide Severity Rating Scale (B-SSRS). | -NSSI history predicted both incident SAs and incident NSSI through week 24.  -NSSI history was a stronger predictor of future attempts than a history of SAs. |
| Aseltine, R. H., James, A., Schilling, E. A., & Glanovsky, J. (2007). Evaluating the SOS suicide prevention program: a replication and extension. *BMC Public Health*, *7*, 161 | Randomized Control Trial | USA | 4133 students in high schools randomly assigned to intervention and control groups. | To examine the effectiveness of the Signs of Suicide (SOS) prevention program in reducing suicidal behavior. | Self-report questionnaires:  -Youth Risk Behavior Survey  The measures of knowledge and attitudes about depression and suicide were adapted from instruments previously used. | Significantly lower rates of suicide attempts and greater knowledge and more adaptive attitudes about depression and suicide were observed among students in the intervention group. |
| Brausch, A. M., & Gutierrez, P. M. (2009).  Differences in Non-Suicidal Self-Injury and Suicide Attempts in Adolescents. *Journal of Youth and Adolescence*, *39*, 233‑242 | Original retrospective case-control study | USA | 373 high school students divided into three groups: no history of self-harm, NSSI only and NSSI + SA. | To examine differences between three groups of adolescents with varying levels of self-harmful behavior (no history of self-harm, NSSI only, NSSI + SA). | Self-report measures:  -Reynolds Adolescent Depression Scale (RADS-2)  -Suicidal Ideation Questionnaire (SIQ)  -Beck Hopelessness Scale (BHS)  -Self-Harm Behavior Questionnaire (SHBQ)  -Rosenberg Self-Esteem Scale (SES)  -Child and Adolescent Social Support Scale (CASSS)  -Eating Attitudes Test (EAT). | -No self-harm group reporting the lowest levels of risk factors and highest levels of protective factors.  - Adolescents in the NSSI group were found to have fewer depressive symptoms, lower suicidal ideation, and greater self-esteem and parental support than the group that also had attempted suicide. |
| Baetens, I., Claes, L., Muehlenkamp, J., Grietens, H., & Onghena, P. (2011). Non-suicidal and suicidal self-injurious behavior among Flemish adolescents: A web-survey.  *Archives of Suicide Research: Official Journal of the International Academy for Suicide Research*, *15*(1), 56‑67 | Original retrospective study | Belgium | 1,417 Flemish adolescents. | To investigate the prevalence of non-suicidal self-injury (NSSI) and suicidal self-injury (SSI) and psychosocial differences between two groups (NSSI and SSI functions, sociodemographic correlates, help seeking behaviors, and stressful life events). | Questionnaires:  -Child and Adolescent Self-Harm in Europe Questionnaire – Dutch Version  -Reasons for Attempting Suicide Questionnaire. | - Lifetime prevalence of NSSI was 13.71% and SSI was 3.93%.  - Significant differences in functions of the behavior, number of stressful life events and likelihood of receiving professional help were noted between groups. |
| Bureau, J.-F., Martin, J., Freynet, N., Poirier, A. A., Lafontaine, M.-F., & Cloutier, P. (2009). Perceived Dimensions of Parenting and Non-suicidal Self-injury in Young Adults. *Journal of Youth and Adolescence*, *39*, 484‑494 | Original retrospective case-control study | Canada | 1238 students in University assigned to Non-NSSI group (n=1133) and NSSI group (n=105). | To identify specific dimensions (failed protection, anger, fear, overcontrol, and feelings of alienation, trust, communication and care) underlying early parent–child relationships in association with NSSI. | Self-report questionnaires:  -Ottawa Self-Injury Inventory  -Adolescent Unresolved Attachment Questionnaire  -Parental Bonding Index  -Inventory of Parent and Peer Attachment. | -Significant differences were found for the relationship dimensions between the two groups.  -When shared variance was accounted for, fear and alienation were the only dimensions predicting NSSI. |
| Cavanagh, J. T. O., Carson, A. J., Sharpe, M., & Lawrie, S. M. (2003).  Psychological Autopsy Studies of Suicide: A Systematic Review. *Psychological Medicine*, *33*(03), 395‑405 | PsychologicalAutopsy Studies:  100 and 54 reports were identified, of which 76 met the criteria for inclusion: 54 case series and 22 case-control studies. | England | N/A | To examine the results of studies of suicide that used a psychological autopsy method. | -Diagnostic and Statistical Manual III, III-R, IV. | -The median proportion of cases with mental disorder was 91%(95%CI 81–98%) in the case series. In the case–control studies the figure was 90% (88–95%) in the cases and 27%(14–48%) in the controls.  -Co-morbid mental disorder and substance abuse also preceded suicide in more cases (38%, 19–57%) than controls (6%, 0–13%).  -The results indicated that mental disorder was the most strongly associated variable of those that have been studied. |
| Connor, J. J., & Rueter, M. A. (2006).  Parent-child relationships as systems of support or risk for adolescent suicidality. *Journal of Family Psychology*, *20*, 143‑155 | Original prospective cohort study | USA | 451 families (with father, mother and a child 12 years old at inclusion). | To examine a process model of predicting adolescent suicidality.  Adolescent emotional distress was hypothesized to mediate the relationship between parental behaviors (warmth or hostility) and subsequent adolescent suicidality. | -Observational assessments of individual, dyadic and family group interactions to evaluate parental warmth and hostility  -Three subscales from the Symptom Checklist-90-Revised (SCL-90-R) to evaluate adolescent emotional distress  -Youth Risk Behavior Survey to evaluate Adolescent suicidality. | -Adolescent emotional distress was found to be a mediating variable between paternal warmth and adolescent suicidality.  -Results indicated that maternal warmth predicted adolescent suicidality but not emotional distress.  -Parental hostility did not predict either latent variable. |
| Cooper, J., Kapur, N., Webb, R., Lawlor, M., Guthrie, E., Mackway-Jones, K., & Appleby, L. (2005).  Suicide after deliberate self-harm: a 4-year cohort study. *The American Journal of Psychiatry*, *162*(2), 297‑303 | Original prospective cohort study | England | 7,968 deliberate self-harm attendees at the emergency departments (adolescents and adults). | -To estimate suicide rates up to 4 years after a deliberate self-harm episode.  -To investigate time-period effects on the suicide rate over the follow-up period (4 years). | Measures of:  -Suicide rates  -Standardized Mortality Ratios. | -Sixty suicides occurred in the cohort during the follow-up period. An approximately 30-fold increase in risk of suicide, compared with the general population, was observed for the whole cohort.  -Suicide rates were highest within the first 6 months after the index self-harm episode. |
| Darche, M. (1990). Psychological factors differentiating self-mutilating and non self-mutilating adolescent inpatient families.  *Journal of Family Psychology*, *21*, 31‑35 | Original retrospective case-control study | USA | 96 patients adolescents: 48 nonsuicidal self-mutilating and 48 nonmutilating. | To compare 48 nonsuicidal **self-mutilating** female psychiatric patients (aged 13–17 yrs) with 48 age- and sex-matched nonmutilating patients using chart reviews of clinical and descriptive characteristics. | **N/A** | **-Self**-**mutilating** were characterized by higher frequencies of sleep disorders, sexual abuse and greater treatment with psychotropic medications.  -They had higher levels of depression, anxiety, hostility, and somatic complaints and greater difficulties with body comfort and confidence.  -They had more Axis 1 diagnoses of affective and eating disorders. |
| Darke, S., Torok, M., Kaye, S., & Ross, J. (2010). Attempted Suicide, Self-Harm, and Violent Victimization among Regular Illicit Drug Users. *Suicide and Life-Threatening Behavior*, *40*(6), 587‑596 | Original retrospective study | Australia | 400 regular users of heroin and/or psychostimulants (adolescents and adults). | To examine relationships among attempted suicide, nonsuicidal self-harm, and physical assault in regular users of heroin and/or psychostimulants. | Structured interview addressed demographics, drug use history, psychological functioning, criminal histories, and personal history of physical violence. | -28% percent had episodes of nonsuicidal self-harm, 32% had attempted suicide, and 95% had been violently assaulted.  -The number of suicide attempts and nonsuicidal self-harm incidents were correlated (ρ = 0.44).  -There were also significant correlations between the number of assaults and nonsuicidal self-harm incidents (ρ = 0.17), and suicide attempts (ρ = 0.27). |
| Dougherty, D. M., Mathias, C. W., Marsh-Richard, D. M., Prevette, K. N., Dawes, M. A., Hatzis, E. S., and al. (2009).  Impulsivity and Clinical Symptoms among Adolescents with Non-Suicidal Self-Injury with or without Attempted Suicide. *Psychiatry research*, *169*(1), 22‑27 | Original experimental study | USA | 56 adolescents recruited from an inpatient psychiatric hospital unit: 25 in NSSI + SA group, 31 in NSSI-Only group. | To examine **clinical** characteristics and laboratory-measured impulsive behavior of **adolescents** engaging in either **non-suicidal self-injury** with (NSSI + SA; *n* = 25) or **without** (NSSI-Only; *n* = 31) **suicide** attempts. | Self-report measures:  -Beck Depression Inventory-II  -Beck Hopelessness Scale  -Beck Scale for Suicidal Ideation  -Barratt Impulsiveness Scale  -Lifetime History of Aggression.  Behavioral assessment of impulsivity:  -Two Choice Impulsivity Paradigm (TCIP)  -GoStop Impulsivity Paradigm (GoStop). | -NSSI + SA patients reported worse depression, hopelessness, and **impulsivity** on standard **clinical** measures, and demonstrated elevated **impulsivity** on a reward-directed laboratory measure compared to NSSI-Only patients. |
| Favaro, A., Santonastaso, P., Monteleone, P., Bellodi, L., Mauri, M., Rotondo, A., and al. (2007).  Self-injurious behavior and attempted suicide in purging bulimia nervosa: Associations with psychiatric comorbidity.  *Journal of Affective Disorders*, *105*(1-3), 285 ‑ 289 | Original retrospective study | Italy | 95 patients with purging type bulimia nervosa (young adults). | To investigate the axis I and II comorbidity in subjects with bulimia nervosa who report self-injurious behavior and/or suicide attempt. | Interviews:  -Structured Clinical Interview for DSM-IV-I (SCID-I) and SCID-II.  Temperament and Character Inventory Revised (TCI-R). | -No axis I diagnosis was associated with any type of self-injurious behavior, whereas social phobia and bipolar disorder were linked to attempted suicide.  -Significant independent predictors of impulsive self-injurious behavior were the presence of childhood sexual abuse, high harm avoidance scores, and high self-transcendence scores, whereas childhood sexual abuse, the presence of a cluster B personality disorder, and a low self-directedness were predictors of suicide attempts. |
| Favazza, A. R. (1998).  The Coming of Age of Self-Mutilation.  *The Journal of Nervous & Mental Disease*, *186*, 259‑268 | Review | USA | N/A | To classify self-mutilations. | N/A | -This review suggests that SM can best be understood as a morbid self-help effort providing rapid but temporary relief from feelings of depersonalization, guilt, rejection, and boredom.  - Favazza classes self-mutilations into three phenomenological categories: major, stereotypic, superficial/moderate (compulsive and impulsive). |
| Firestone, R. W., & Seiden, R. H. (1990).  Suicide and the continuum of self-destructive behavior. *Journal of American College Health: J of ACH*, *38*(5), 207‑213 | Theoretical paper | USA | N/A | -Describe a "voice" process (VP) by which **suicide** and **self-destructive behavior** (SDB) are influenced.  - Understanding where an individual can be placed on the continuum of self-destructive thoughts and actions can assist clinicians in their diagnoses. | N/A | -The authors provide a chart depicting the levels of increasing suicidal intent along the continuum.  -The chart identifies specific negative thoughts and injunctions typically reported by persons who attempt suicide, neurotic patients, and "normal" subjects. |
| Franklin, J. C., Hessel, E. T., & Prinstein, M. J. (2011). Clarifying the role of pain tolerance in suicidal capability.  *Psychiatry Research*, *189*(3), 362‑367 | Original experimental study | USA | 67 undergraduates. | Investigate associations among painful and provocative events, nonsuicidal self-injury, acquired capability for suicide, and pain tolerance, threshold, and perceived intensity. | Questionnaires:  -Painful and Provocative Events Scale (PPE Scale)  -Functional Assessment of Self-Mutilation (FASM)  -Acquired Capability for Suicide Scale (ACS).  Experimental task:  -Cold pressor task: measures of pain threshold, tolerance and pain intensity ratings. | -Results were highly consistent with the Joiner’s interpersonal-psychological theory of suicide.  -Results also supported the interpersonal-psychological theory hypothesis that nonsuicidal self-injury represents an important painful and provocative event that increases suicidal capability. Specifically, participants with a history of nonsuicidal self-injury displayed increased suicidal capability and decreased pain perception. |
| Glenn, C. R., & Klonsky, E. D. (2009).  Social context during non-suicidal self-injury indicates suicide risk.  *Personality and Individual Differences*, *46*(1), 25‑29 | Original retrospective study | USA | 205 students who endorsed having engaged in at least one form of NSSI. | To examine the relationship among automatic/ intrapersonal functions, social context during NSSI (i.e., the extent to which one self-injures alone versus around others), and suicidality. | Self-report measures:  -Youth Risk Behaviors Survey (YRBS)  -McLean Screening Instrument for Borderline Personality Disorder (MSI-BPD)  -Depression Anxiety Stress Scales (DASS-21)  -Inventory of Statements About Self-Injury (ISAS). | -Self-injurers who engage in NSSI alone were more likely to report a history of suicide ideation, plans, and attempts compared to other self-injurers.  -Social context during NSSI appears to be a marker for suicide risk in individuals who engage in NSSI. |
| Glenn, J. J., Michel, B. D., Franklin, J. C., Hooley, J. M., & Nock, M. K. (2014). Pain analgesia among adolescent self-injurers. *Psychiatry Research*, *220*(3), 921‑926 | Original experimental case-control study | USA | 79 adolescents recruited from the community: 58 case (adolescent self-injurers) and 21 non-injurious controls. | To test:  -whether the pain analgesia effects observed among adult self-injurers are also present among adolescents.  -three potential explanatory models proposing that habituation, dissociation, and/or self-criticism help explain the association between NSSI and pain analgesia among adolescents. | Interviews:  -Kiddie Schedule for Affective Disorders and Schizophrenia for School Age Children-Present and Lifetime Version (K-SADS-PL)  -Adolescent Dissociative Experiences Scale-II (A-DES)  -Self-Injurious Thoughts and Behaviors Interview (SITBI)  -Self-Rating Scale (SRS).  Task:  -Finger pressure algometer to measure perception of physical pain. | - Adolescent self-injurers have a higher pain threshold and greater pain endurance than non-injurers.  - A self-critical style does mediate the association between NSSI and pain analgesia. |
| Guertin, T., Lloyd-Richardson, E., Spirito, A., Donaldson, D., & Boergers, J. (2001).  Self-mutilative behavior in adolescents who attempt suicide by overdose.  *Journal of the American Academy of Child and Adolescent Psychiatry*, *40*(9), 1062‑1069 | Original retrospective study | USA | 95 adolescents evaluated after a suicide attempt that occurred between 1996 and 2000 were divided into two groups: suicide attempters with a history of SMB (n = 52) and suicide attempters without SMB (n = 43). | To examine the cognitive/affective and behavioral symptoms of adolescent suicide attempters with self-mutilative behavior (SMB). | Self-report measures:  -Functional Assessment of Self-Mutilation (FASM)  Questionnaires:  -Diagnostic Interview Schedule for Children (NIMH-DISC), Suicide Intent Scale, Center for Epidemiologic Studies-Depression Scale (CES-D), Hopelessness Scale for Children (HSC), Revised UCLA Loneliness Scale (UCLA-LS), State-Trait Anger Expression Inventory (STAXI), Adolescent Risk Taking Scale (ARTS), Reckless Behavior Questionnaire (RBQ), Adolescent Drinking Index (ADI) and McMaster Family Assessment Device (FAD). | -The SMB group was significantly more likely to be diagnosed with oppositional defiant disorder, major depression, and dysthymia and had higher scores on measures of hopelessness, loneliness, anger, risk taking, reckless behavior, and alcohol use than did the non-SMB group. |
| Hamza, C. A., Stewart, S. L., & Willoughby, T. (2012). Examining the link between nonsuicidal self-injury and suicidal behavior: A review of the literature and an integrated model.  *Clinical Psychology Review*, *32*(6), 482‑495 | Systematic review | Canada | N/A | Comprehensive review of the literature on link between NSSI and suicidal behavior. | N/A | -We summarize several studies that specifically examined the association between NSSI and suicidal behavior.  -Three theories that have been proposed to account for the link between NSSI and suicidal behavior are described, and the empirical support for each theory is critically examined. |
| Hargus, E., Hawton, K., & Rodham, K. (2009). Distinguishing Between Subgroups of Adolescents Who Self‐Harm.  *Suicide and Life-Threatening Behavior*, *39*(5), 518‑537 | Original retrospective study | England | 6,020 adolescents aged 15 and 16 years. | Investigate the differences in factors associated with subgroups of adolescents in the continuum of deliberate self-harm (DSH) phenomena. | Self-report measures:  -Robson Self-Concept Questionnaire  -Plutchik and Van Praag Scale  -Hospital Anxiety and Depression Scale. | -3.2% of adolescents reported DSH with intent to die, 2.8% reported DSH without intent to die, and 15% reported thoughts of DSH without acts.  -Familial and non-familial social influences on DSH behavior: a uniquely distinct relationship was found between DSH of a friend and DSH without intent to die on one hand and DSH of a family member with DSH with intent to die on the other. |
| Hawton, K., Cole, D., O’Grady, J., & Osborn, M. (1982).  Motivational aspects of deliberate self-poisoning in adolescents.  *The British Journal of Psychiatry*, *141*, 286‑291 | Original retrospective study | England | 50 adolescent self-poisoners. | Demonstrate considerable discrepancies between the reasons chosen by the subjects to explain the overdoses and those chosen by clinical assessors. | N/A | -Most adolescents indicated that they had been feeling lonely or unwanted, or angry with someone, and had taken the overdose to alleviate or demonstrate this distress. A third said they had wanted to die.  - In contrast, clinical assessors tended to attribute the overdose to punitive or manipulative reasons and suggested that only seven out of the 50 had wished to die.  -Adolescents rarely indicated that they had taken the overdose to get help. |
| Hawton, K., Saunders, K. E. A., & O’Connor, R. C. (2012).  Self-harm and suicide in adolescents.  *Lancet*, *379*(9834), 2373‑2382 | Theoretical paper in a Series of papers about suicide | England | N/A | Summary of self-harm and suicide characteristics in adolescents. | N/A | -Study of epidemiology, factors associated with self-harm and suicide.  -Listing of key challenges to prevention of self-harm and suicide in adolescents.  -Summary of psychosocial interventions and pharmacotherapy. |
| Hilt, L. M., Nock, M. K., Lloyd-Richardson, E. E., & Prinstein, M. J. (2008). Longitudinal Study of Nonsuicidal Self-Injury Among Young Adolescents Rates, Correlates, and Preliminary Test of an Interpersonal Model.  *The Journal of Early Adolescence*, *28*(3), 455‑469 | Original longitudinal cohort study | USA | Sample of 508 sixth, seventh, and eighth graders. | To examine **rates, correlates** and an **interpersonal model** of **nonsuicidal self-injury** (NSSI). | Administered two times over an 11-month period:  -Youth Risk Behaviors Survey (YRBS)  -Inventory of Parent and Peer Attachment (IPPA). | -7.5% reported engaging in NSSI within the past year with no significant differences across genders, ethnicities, or grade.  -Those engaging in NSSI were more likely to report having smoked cigarettes, taken drugs, and engaged in maladaptive eating behaviors.  -Consistent with an **interpersonal model**, those engaging in NSSI reported significant increases in the quality of their relationships with fathers over time. |
| Hooley, J. M., Ho, D. T., Slater, J., & Lockshin, A. (2010).  Pain perception and nonsuicidal self-injury: a laboratory investigation. *Personality Disorders*, *1*(3), 170‑179 | Original experimental case-control study | USA | 67 young adults recruited from the community divided into two groups: 31 in self-injury group, 36 in control group. | To examine group differences in pain threshold and pain endurance between participants who self-injured and control participants who were exposed to pressure pain applied to the finger. | Self-report measure:  -Locus of Control of behavior Scale (LCB)  Questionnaires:  -Dissociation Experiences Scale (DES)  -NEO Five Factor Inventory (NEO-FFI)  -Hopelessness Scale.  Task:  -Pressure algometer to measure physical pain. | -Participants who self-injured had higher pain thresholds (time to report pain) and endured pain for longer than control participants.  -Among participants who self-injured, those with longer histories of self-injury had higher pain thresholds.  -A highly self-critical cognitive style was the strongest predictor of prolonged pain endurance. |
| Hukkanen, R., Sourander, A., & Bergroth, L. (2003). Suicidal ideation and behavior in children’s homes.  *Nordic Journal of Psychiatry*, *57*(2), 131‑137 | Original retrospective study | Finland | 98 children and adolescents in child welfare institutions. | To report the self-destructive and suicidal behavior of children and adolescents in child welfare institutions. | Questionnaires:  -Spectrum of Suicidal Behavior Scale  -Child Behavior Checklist (CBCL)  -Children’s Global Assessment Scale (CGAS)  -Spectrum of Assaultive Behavior Scale. | -Thirty-two per cent of the sample had presented suicidal thoughts, threats or suicide attempts during the previous 6 months.  -Suicidality was associated with low general functioning level (CGAS<61), self-mutilating behavior and violence.  -Suicidal children had significantly higher CBCL total, externalizing, internalizing, anxious-depressive and aggressive scores.  -Children with suicide attempts (8% of the sample) had a significantly higher number of different types of traumatic experiences before the placement and higher somatization syndrome scores compared to children with suicidal ideation or non-suicidal children. |
| Jacobson, C. M., Muehlenkamp, J. J., Miller, A. L., & Turner, J. B. (2008). Psychiatric impairment among adolescents engaging in different types of deliberate self-harm.  *Journal of Clinical Child and Adolescent Psychology: The Official Journal for the Society of Clinical Child and Adolescent Psychology, American Psychological Association, Division 53*, *37*(2), 363‑375 | Retrospective chart review study | England | 227 adolescents divided into four groups: no deliberate self-harm (NoDSH; n = 119), nonsuicidal self-injury only (NSSI only; n = 30), suicide attempt only (n = 38), and suicide attempt plus NSSI (n = 40). | To examine the psychiatric profiles of outpatient adolescents engaging in different types of deliberate self-harm (DSH) behaviors. | Semi-structured interviews:  -Lifetime Parasuicide Count (LPC)  -Schedule for Affective Disorders and Schizophrenia for school-aged children (K-SADS)  -Structured Clinical Interview for DSM-IV Personality Disorders Questionnaire, Borderline Personality Disorder Module (SCID-II BPD)  Self-report measures:  -Beck Depression Inventory II (BDI-II)  -Suicidal Ideation Questionnaire (SIQ). | -Those who attempted suicide were more likely to have major depressive disorder and/or posttraumatic stress disorder than those who engaged in NSSI only.  -Those who engaged in any type of DSH were more likely to have features of borderline personality disorder than those who did not engage in DSH.  -The suicidal ideation levels of those in the NSSI group were similar to those in the NoDSH group. |
| Klonsky, E. D., & Glenn, C. R. (2008).  Assessing the Functions of Non-suicidal Self-injury: Psychometric Properties of the Inventory of Statements About Self-injury (ISAS). *Journal of Psychopathology and Behavioral Assessment*, *31*(3), 215‑219 | Original retrospective study | USA | 235 young adults from a college population who had performed at least one NSSI behavior. | To report the psychometric properties of the Inventory of Statements About Self-injury (ISAS), a measure designed to comprehensively assess the functions of non-suicidal self-injury (NSSI). | Questionnaires:  -Inventory of Statements About Self-Injury (ISAS)  -Youth Risk Behaviors Survey (YRBS)  Self-report measures:  -Depression Anxiety Stress Scales (DASS-21)  -McLean Screening Instrument for Borderline Personality Disorder (MSI-BPD). | -ISAS functions comprised two factors representing interpersonal and intrapersonal functions.  -ISAS factors exhibited excellent internal consistency and expected correlations with both clinical constructs (e.g., borderline personality disorder, suicidality, depression, anxiety) and contextual variables.  -The ISAS may be useful in research and treatment contexts as a comprehensive measure of NSSI functions. |
| Lachal, J., Orri, M., Sibeoni, J., Moro, M. R., & Revah-Levy, A. (2015). Metasynthesis of youth suicidal behaviours: perspectives of youth, parents, and health care professionals.  *PloS One*, *10*(5), e0127359 | Systematic review of qualitative studies | France | N/A | To synthesise the views of suicidal adolescents and young adults, their parents, and their healthcare professionals on the topics of suicidal behaviour and management of those who have attempted suicide. | N/A | The violence of the message of a suicidal act and the fears associated with death lead to incomprehension and interfere with the capacity for empathy of both family members and professionals. |
| Muehlenkamp, J. J., Claes, L., Havertape, L., & Plener, P. L. (2012).  International prevalence of adolescent non-suicidal self-injury and deliberate self-harm.  *Child and Adolescent Psychiatry and Mental Health*, *6*(1), 1‑9 | Systematic review | USA | N/A | Review of current (2005 - 2011) empirical studies reporting on the prevalence of NSSI and DSH in adolescent samples across the globe. | N/A | -No statistically significant differences were found between NSSI and DSH studies.  - Mean prevalence rates have not increased in the past five years, suggesting stabilization. |
| Muehlenkamp, J. J., Ertelt, T. W., Miller, A. L., & Claes, L. (2011).  Borderline personality symptoms differentiate non-suicidal and suicidal self-injury in ethnically diverse adolescent outpatients. *Journal of Child Psychology and Psychiatry*, *52*(2), 148‑155 | Retrospective chart review study | USA | 441 ethnically diverse adolescents. | To examine how specific borderline personality disorder (BPD) symptoms relate to suicide attempts or suicide and non-suicidal self-injury (NSSI) within adolescent populations. | Semi-structured interviews:  -Lifetime Parasuicide Count (LPC)  -Structured Clinical Interview for DSM-IV Personality Disorders Questionnaire: Borderline Personality Disorder Module (SCID-II-BPD)  -Schedule for Affective Disorders and Schizophrenia for school-aged children (K-SADS)  Self-report measure:  -Life Problems Inventory (LPI). | Two BPD symptoms (confusion about self and unstable interpersonal relationships) exhibit distinct relationships to NSSI and suicide attempts, but there is not strong variation in their relationship to BPD. |
| Muehlenkamp, J. J., & Gutierrez, P. M. (2004).  An Investigation of Differences Between Self-Injurious Behavior and Suicide Attempts in a Sample of Adolescents. *Suicide and Life-Threatening Behavior*, *34*(1), 12‑23 | Original retrospective study | USA | 390 high school students. | To examine potential differences between adolescents who had attempted suicide and those who engaged in self-injurious behavior on measures of depression, suicidal ideation, and attitudes toward life and death. | Self-report measures:  -Suicidal Ideation Questionnaire (SIQ)  -Reynolds Adolescent Depression Scale (RADS)  Questionnaires:  -Multi-Attitude Suicide Tendency Scale (MAST)  -Self-Harmful Behavior Scale (SHB). | -A significant difference on attitudes toward life was found between the self-injury and suicide attempt groups.  -Measures of depression, suicide ideation, and attitudes towards life predicted participants' self-harm categorization. |
| Muehlenkamp, J. J., & Gutierrez, P. M. (2007).  Risk for Suicide Attempts Among Adolescents Who Engage in Non-Suicidal Self-Injury.  *Archives of Suicide Research*, *11*(1), 69‑82 | Original retrospective study | USA | 540 high school students. | To examine whether common indicators of suicide risk differ between adolescents engaging in non-suicidal self-injury (NSSI) who have and have not attempted suicide. | Self-report questionnaires:  -Reasons for Living Inventory for Adolescents (RFL-A)  -Reynolds Adolescent Depression Scale (RADS)  -Suicidal ideation Questionnaire (SIQ)  -Self-Harm Behavior Questionnaire (SHBQ). | Adolescents engaging in NSSI who also attempt suicide can be differentiated from adolescents who only engage in NSSI on measures of suicidal ideation, reasons for living, and depression. |
| Muehlenkamp, J. J., Walsh, B. W., & McDade, M. (2009).  Preventing Non-Suicidal Self-Injury in Adolescents: The Signs of Self-Injury Program.  *Journal of Youth and Adolescence*, *39*(3), 306‑314 | Original prospective longitudinal study | USA | 274 adolescents of five schools implemented the program. | To evaluate the signs of self-injury program who is the first known NSSI school-based prevention program for adolescents. | Pre-post evaluation surveys of the program.  Self-report questionnaires:  -Self-Injurious Thoughts and Behaviors Inventory (SITBI)  Items inquiring about knowledge of NSSI and attitudes towards help-seeking were adapted from Aseltine and DeMartino’s survey (Aseltine, R.H., & DeMartino, R. (2004). An outcome evaluation of the SOS suicide prevention program. American Journal of Public Health, 94, 446-451). | -The prevention program did not produce iatrogenic effects, increased accurate knowledge and improved help-seeking attitudes and intentions among students.  -No significant changes were found in regards to self-reported formal help-seeking actions.  - The data offer preliminary evidence that the program may be an effective prevention program for schools. |
| Nock, M. K., Joiner, T. E., Gordon, K. H., Lloyd-Richardson, E., & Prinstein, M. J. (2006).  Non-suicidal self-injury among adolescents: diagnostic correlates and relation to suicide attempts. *Psychiatry Research*, *144*(1), 65‑72 | Original retrospective study | USA | 89 adolescents admitted to an adolescent psychiatric inpatient unit who engaged in NSSI in the previous 12 months. | To examine diagnostic correlates of adolescents with a recent history of NSSI and the relation between NSSI and suicide attempts. | Clinical interviews:  -Diagnostic Interview Schedule for Children (DISC)  -Diagnostic Interview for DSM-IV Personality Disorders (DIPD-IV)  Self-report measure:  -Functional Assessment of Self-Mutilation (FASM). | - 87.6% of adolescents engaging in NSSI met criteria for a DSM-IV Axis I diagnosis and 67.3% met criteria for an Axis II personality disorder.  - 70% of adolescents engaging in NSSI reported a lifetime suicide attempt and 55% reported multiple attempts. |
| Nock, M. K., Borges, G., Bromet, E. J., Cha, C. B., Kessler, R. C., & Lee, S. (2008).  Suicide and Suicidal Behavior.  *Epidemiologic Reviews*, *30*(1), 133‑154 | Systematic review | USA | N/A | To examine the prevalence of, trends in, and risk and protective factors for suicidal behavior in the United States and cross-nationally. | N/A | -The data revealed significant cross-national variability in the prevalence of suicidal behavior but consistency in age of onset, transition probabilities, and key risk factors.  -Suicide is more prevalent among men, whereas nonfatal suicidal behaviors are more prevalent among women and persons who are young, are unmarried, or have a psychiatric disorder. |
| Nock, M. K., & Prinstein, M. J. (2004).  A functional approach to the assessment of self-mutilative behavior.  *Journal of Consulting and Clinical Psychology*, *72*(5), 885‑890 | Original retrospective study | USA | 108 adolescent psychiatric inpatients referred for self-injurious thoughts or behaviors. | This study applied a functional approach (automatically reinforcing and/or socially reinforcing properties) to the assessment of self-mutilative behavior (SMB) among adolescent psychiatric inpatients. | Self-report measure:  -Functional Assessment of Self-Mutilation (FASM). | -Adolescents reported engaging in SMB frequently, using multiple methods, and having an early age of onset.  -The results supported the structural validity and reliability of the hypothesized functional model of SMB. Most adolescents engaged in SMB for automatic reinforcement, although a sizable portion endorsed social reinforcement functions as well. |
| Nock, M. K., & Prinstein, M. J. (2005).  Contextual features and behavioral functions of self-mutilation among adolescents.  *Journal of Abnormal Psychology*, *114*(1), 140‑146 | Original retrospective study | USA | 89 adolescent psychiatric inpatients. | To examine the contextual features and behavioral functions of self-mutilative behavior (SMB) in a sample of adolescents. | Self-report measure:  -Functional Assessment of Self-Mutilation (FASM)  Structured Clinical Interview:  -Diagnostic Interview Schedule for Children (DISC). | -SMB typically was performed impulsively, in the absence of physical pain, and without the use of alcohol or drugs.  -Moreover, analyses supported the construct validity of a functional model in which adolescents reported engaging in SMB for both automatic and social reinforcement.  - Recent suicide attempt and hopelessness were associated with only  the automatic negative reinforcement function of SMB. |
| Nock, M. K., Prinstein, M. J., & Sterba, S. K. (2009). Revealing the form and function of self-injurious thoughts and behaviors: A real-time ecological assessment study among adolescents and young adults.  *Journal of Abnormal Psychology*, *118*(4), 816‑827 | Original prospective study | USA | 30 **adolescents** and **young adults** with a recent history of **self**-injury. | To measure suicidal and nonsuicidal SITBs as they naturally occur in **real time** by using **ecological** momentary **assessment** methods. | Interviews:  -Self-Injurious Thoughts and Behaviors Interview (SITBI)  - Schedule for Affective Disorders and Schizophrenia for School-Aged Children (K-SADS)  Adolescents completed signal- and event-contingent assessments, handheld computers. Participants responded to a brief structured series of multiple-choice questions at each data entry period about the form and functions of SITBs. | -Participants reported an average of 5.0 **thoughts** of nonsuicidal **self**-injury (NSSI) per week, most often of moderate intensity and short duration (1–30 min), and 1.6 episodes of NSSI per week.  -Suicidal **thoughts** occurred less frequently (1.1 per week), were of longer duration, and led to suicide attempts less often. |
| Plener, P. L., Libal, G., Keller, F., Fegert, J. M., & Muehlenkamp, J. J. (2009). An international comparison of adolescent non-suicidal self-injury (NSSI) and suicide attempts: Germany and the USA.  *Psychological Medicine*, *39*(09), 1549–1558 | Original retrospective study | Germany USA | 665 adolescents in a school setting. | To examine the prevalence of non-suicidal self-injury (NSSI), suicide attempts, suicide threats and suicidal ideation in a German school sample and compared the rates with a similar sample of adolescents from the midwestern USA. | Self-report questionnaires:  -Ottawa Self-Injury Inventory (OSI)  -German version of Center for Epidemiologic Studies-Depression Scale (CES-D)  -Self-Harm Behavior Questionnaire (SHBQ). | -25,6% of the participants endorsed at least one act of NSSI in their life, and 9.5% of those students answered that they had hurt themselves repetitively. 6.5% of the students reported a history of a suicide attempt.  -No differences were found in the prevalence and characteristics of self-injury and suicidal behaviors between adolescents from Germany and the USA. |
| Prinstein, M. J., Nock, M. K., Simon, V., Aikins, J. W., L, S., & Spirito, A. (2008). Longitudinal trajectories and predictors of adolescent suicidal ideation and attempts following inpatient hospitalization.  *Journal of Consulting and Clinical Psychology*, *76*(1), 92‑103 | Original prospective cohort study | USA | A sample of 143 **adolescents** was assessed during psychiatric **inpatient hospitalization** and again at 3, 6, 9, 15, and 18 months post-discharge. | To assess the temporal course of **adolescent suicidal ideation** and behavior. | Questionnaires:  -Suicidal Ideation Questionnaire (SIQ)  -Kiddie-Schedule for Affective Disorders and Schizophrenia  -National Institute of Mental Health Diagnostic Interview Schedule for Children (NIMH-DISC-C) and for parents (NIMH-DISC-P)  -Youth Risk Behavioral Surveillance System  -Children’s Depression Inventory (CDI)  Self-report interview:  -Self-Reported Delinquency Interview. | -Higher **adolescent**-reported depressive symptoms, lower parent-reported externalizing symptoms, and higher frequencies of NSSI predicted weaker **suicidal ideation** remission slopes.  - Analyses revealed a period of **suicidal ideation** remission between baseline and 6 months **following** discharge, as well as a subtle period of **suicidal ideation** reemergence between 9 and 18 months post-discharge. |
| Ross, S., & Heath, N. (2002). A Study of the Frequency of Self-Mutilation in a Community Sample of Adolescents.  *Journal of Youth and Adolescence*, *31*(1), 67‑77 | Original retrospective study | Canada | 440 students from 2 high schools. | To examine epidemiological data concerning the **frequency** of SM in a **community sample** of high schools students. | Self-report measures:  -Beck Depression Inventory (BDI)  -Beck Anxiety Inventory | -Based on interviews it was found that 13.9% of all students reported having engaged in SM behavior at some time.  - Students who **self**-mutilate reported significantly more anxiety and depressive symptomatology than students who did not **self**-mutilate.  -Girls reported significantly higher rates of SM than did boys. **Self**-cutting was found to be the most common type of SM. |
| Scoliers, G., Portzky, G., Madge, N., Hewitt, A., Hawton, K., de Wilde, E. J., and al. (2009).  Reasons for adolescent deliberate self-harm: a cry of pain and/or a cry for help? Findings from the child and adolescent self-harm in Europe (CASE) study.  *Social Psychiatry and Psychiatric Epidemiology*, *44*(8), 601‑607 | Original retrospective study | Europe, Australia. | 30,477 school pupils between the ages of 14–17 in seven countries. | To examine **reasons** for **adolescent deliberate self**-**harm**. | Questionnaires:  -Lifestyle questionnaire  -Coping questionnaire. | -The majority of **self**-harmers reported at least one **cry** of **pain** motive (‘to die’, ‘to punish myself’, and ‘to get relief from a terrible state of mind’) and an additional **cry** for **help** motive (‘to show how desperate I was feeling’, to frighten someone’, ‘to get my own back on someone’, ‘to find out whether someone really loved me’, and ‘to get some attention’).  -The results showed that ‘wanted to get relief from a terrible state of mind’ and ‘wanted to die’ were most commonly reported. |
| Sinclair, J., & Green, J. (2005).  Understanding resolution of deliberate self harm: qualitative interview study of patients’ experiences.  *BMJ (Clinical Research Ed.)*, *330*(7500), 1112 | Original qualitative in-depth interview study | England | 20 participants (adolescents and adults) selected from a representative cohort after an episode of **deliberate self-harm.** | To understand how those with a history of **deliberate self-harm** perceive this **resolution** and to identify potential implications for provision of health services. | -Principles of grounded theory for thematic analysis  -Frank’s model of illness narratives for narrative analysis. | We identified three recurrent themes: the **resolution** of adolescent distress; the recognition of the role of alcohol as a precipitating and maintaining factor in **self harm**; and the **understanding** of **deliberate self harm** as a symptom of untreated or unrecognised illness. |
| Skegg, K. (2005).  Self-harm.  *Lancet*, *366*(9495), 1471‑1483 | Theoretical paper (Seminar) | New Zealand | N/A | General characteristics of self-harm. | N/A | -Cultural aspects of some societies may protect against suicide and **self**-**harm** and explain some of the international variation in rates of these events.  -Risk of repetition of **self**-**harm** and of later suicide is high.  -More than 5% of people who have been seen at a hospital after **self**-**harm** will have committed suicide within 9 years.  -Strong suicidal intent, high lethality, precautions against being discovered, and psychiatric illness are indicators of high suicide risk. |
| Stanley, B., Gameroff, M. J., Michalsen, V., & Mann, J. J. (2001).  Are suicide attempters who self-mutilate a unique population?  *The American Journal of Psychiatry*, *158*(3), 427‑432 | Original retrospective case-control study | USA | Two groups of young adults:  -One of 30 suicide attempters with cluster B personality disorders who had a history of self-mutilation  -A matched group of 23 suicide attempters with cluster B personality disorders who had no history of self-mutilation. | The purpose of this study was to determine differences between suicide attempters with and without a history of self-mutilation. | Questionnaires:  -Suicide Intent Scale  -Suicide Ideation Scale  -Brown-Goodwin Lifetime Aggression Scale  -Hamilton Depression Rating Scale and Beck Hopelessness Scale  Interviews:  -Schedule for Affective Disorders and Schizophrenia  -Schedule for Interviewing Borderlines. | -Self-mutilators perceived their suicide attempts as less lethal, with a greater likelihood of rescue and with less certainty of death.  -Self-mutilators had more persistent suicide ideation, and their pattern for suicide was similar to their pattern for self-mutilation, which was characterized by chronic urges to injure themselves. |
| Suyemoto, K. L. (1998).  The functions of self-mutilation.  *Clinical Psychology Review*, *18*(5), 531‑554 | Review | USA | N/A | This **review** uses six functional models extracted from the literature to organize a discussion of the multiple **functions** of **self**-**mutilation.** | N/A | -Six functional models are then presented: the environmental model, the anti-suicide model, the sexual model, the affect regulation model, the dissociation model, and the boundaries model. |
| Tang, J., Yu, Y., Wu, Y., Du, Y., Ma, Y., Zhu, H., and al. (2011).  Association between non-suicidal self-injuries and suicide attempts in Chinese adolescents and college students: a cross-section study.  *PloS One*, *6*(4), e17977 | Original retrospective study | China | 2013 Chinese students with the age ranging between 10 and 24 years. (adolescents and young adults). | To examine the association between non-suicidal self-injury (NSSI) and suicide attempts among Chinese adolescents and college students. | Self-report questionnaires:  -Functional Assessment of Self-Mutilation (FASM)  -Center for Epidemiological Studies Depression Scale (CES-D). | -Self-reported prevalence rates of NSSI, suicidal ideation, suicide attempts were 15.5%, 8.8%, and 3.5%, respectively.  -NSSI was significantly associated with self-reported suicide attempts.  -NSSI was significantly associated with greater risk of suicide attempts in those not reporting suicidal ideation than those reporting suicidal ideation in the past year. |
| Timson, D., Priest, H., & Clark-Carter, D. (2012). Adolescents who self-harm: professional staff knowledge, attitudes and training needs. *Journal of Adolescence*, *35*(5), 1307‑1314 | Original retrospective study | England | 120 qualified professionals working within an Accident and Emergency (A&E) Department, Child & Adolescent Mental Health Services (CAMHS) and a Secondary School. | To investigate professional staff attitudes and knowledge about adolescents who engage in self-harming behavior and to identify training needs. | Self-report questionnaires:  -Jeffery and Warm measures of staff knowledge about self-harming (Jeffery, D., & Warm, A. (2002). A study of service providers’ understanding of self-harm. Journal of Mental Health, 11(3), 295–303)  -Crawford measure of staff attitudes to deliberate self-harm (Crawford, T., Geraghty, W., Street, K., & Simonoff, E. (2003). Staff knowledge and attitudes towards deliberate self-harm in adolescents. Journal of  Adolescence, 26, 619–629). | -CAMHS staff were more knowledgeable and felt more effective than either A&E staff or teachers, whereas A&E staff expressed more negative attitudes. |
| Tuisku, V., Kiviruusu, O., Pelkonen, M., Karlsson, L., Strandholm, T., & Marttunen, M. (2014). Depressed adolescents as young adults - predictors of suicide attempt and non-suicidal self-injury during an 8-year follow-up.  *Journal of Affective Disorders*, *152-154*, 313‑319 | Original prospective longitudinal study | Finland | 63.8% of the original study population (n=139) (depressed adolescent outpatients), assessed at baseline, at 1-year and 8-year follow ups. | To study SA and NSSI as predictors of future NSSI and SA, and to study the role of other risk and protective factors. | Diagnostic interview:  -Schedule for Affective Disorders and Schizophrenia for School-Aged Children (K-SADS)  Self-report measures:  -Beck Depression Inventory (BDI-21)  -Perceived Social Support Scale-Revised (PSSS-R)  -Alcohol Use Disorders Identification Test (AUDIT)  Self-report measures:  -Beck Anxiety Inventory (BAI-21). | -SAs were predicted both in the 1-year follow-up and in the period between the 1- and 8-year follow-ups by alcohol use and low perceived peer support.  -NSSI in the 1-year of follow-up was predicted by baseline NSSI, younger age and alcohol use, whereas the only significant predictor for NSSI between the 1- and 8-year follow-ups was NSSI.  -Among depressed outpatients NSSI is a strong predictor of suicidal behavior. |
| Tulloch, A. L., Blizzard, L., & Pinkus, Z. (1997). Adolescent-parent communication in self-harm. *The Journal of Adolescent Health: Official Publication of the Society for Adolescent Medicine*, *21*(4), 267‑275 | Original retrospective case-control study | Australia | 56 adolescents presenting to the accident and emergency department of a general hospital and 52 hospital-based controls. | To examine the association between communication with parents and self-harm in 14-19-year-old adolescents. | Questionnaires:  -Parent-Adolescent Communication Scale (PACS)  -Family Adaptability and Cohesion Evaluation Scale (FACES)  -Adolescent-Family Inventory of Life Events and Changes Scales  -Children’s Depression Index (CDI)  -Nowicki-Strickland Locus of Control (LOC) Scale. | -The absence of a family confidant was very strongly associated with adolescent self-harm.  -Poorer parent-adolescent communication remained strongly associated with self-harm. |
| Turner, B. J., Layden, B. K., Butler, S. M., & Chapman, A. L. (2013).  How often, or how many ways: clarifying the relationship between non-suicidal self-injury and suicidality.  *Archives of Suicide Research: Official Journal of the International Academy for Suicide Research*, *17*(4), 397‑415 | Original prospective longitudinal study | Canada | 142 participants who engaged in NSSI (adolescents and young adults). | To examine whether greater frequency or greater versatility of several self-damaging behaviors, including non-suicidal self-injury (NSSI), substance use, and disordered eating, increased risk for suicide. | Questionnaires at baseline and 3-months later:  -Suicidal Behavior Questionnaire – Revised  -Personality Assessment Inventory – Borderline Scale  -General Severity Index  -Beck Depression Inventory-II  -Personality Assessment Inventory  -Addiction Severity Index  -Beck Hopelessness Scale  -Brief Symptom Inventory  -Eating Disorder Diagnostic Scale  -Deliberate Self-Harm Inventory. | -Results suggest that the versatility rather than frequency of self-damaging behaviors is most robustly associated with suicide risk.  -Versatility of NSSI interacted with depression to predict suicide risk at 3-month follow-up (highly depressed participants who engaged in more methods of NSSI exhibited highest risk). |
| Van Orden, K. A., Witte, T. K., Cukrowicz, K. C., Braithwaite, S. R., Selby, E. A., & Joiner, T. E. (2010). The interpersonal theory of suicide.  *Psychological Review*, *117*(2), 575‑600 | Systematic review and theoretical paper | USA | N/A | To present the interpersonal theory of suicidal behavior. | N/A | -The capability to engage in suicidal behavior is separate from the desire to engage in suicidal behavior.  -The capability for suicidal behavior emerges, via habituation and opponent processes, in response to repeated exposure to physically painful and/or fear-inducing experiences. |
| Van Orden, K. A., Witte, T. K., Gordon, K. H., Bender, T. W., & Joiner Jr., T. E. (2008).  Suicidal desire and the capability for suicide: Tests of the interpersonal-psychological theory of suicidal behavior among adults.  *Journal of Consulting and Clinical Psychology*, *76*(1), 72‑83 | Three original retrospective studies | USA | -Study 1: 309 undergraduate students.  -Study 2: 228 outpatients (young adults).  -Study 3: 153 outpatients (young adults). | To test the theory's hypotheses. | -Study 1: Interpersonal Needs Questionnaire (INQ), Beck Scale for Suicide Ideation (BSS), Beck Depression Inventory (BDI)  -Study 2: BSS, BDI, Impulsive Behavior Scale (IBS), Painful and Provocative Events Scale (PPES), Acquired Capability for Suicide Scale (ACSS)  -Study 3: BDI, INQ, ACSS, Clinician ratings of suicide risk. | -In Study 1, the interaction of thwarted belongingness and perceived burdensomeness predicted current suicidal ideation.  -In Study 2, greater levels of acquired capability were found among individuals with greater numbers of past attempts.  -In Study 3, the interaction of acquired capability and perceived burdensomeness predicted clinician-rated risk for suicidal behavior. |
| Victor, S. E., & Klonsky, E. D. (2014).  Correlates of suicide attempts among self-injurers: a meta-analysis.  *Clinical Psychology Review*, *34*(4), 282‑297 | Meta-analysis | Canada | N/A | Examining predictors of SA history among self-injurers. | N/A | -The strongest correlate of SA history was suicidal ideation.  -After suicidal ideation, the strongest predictors of SA history were NSSI frequency, number of NSSI methods, and hopelessness. |
| Whitlock, J. (2010).  Self-injurious behavior in adolescents.  *PLoS Medicine*, *7*(5), e1000240 | Theoretical paper | USA | N/A | To make a point on the situation: NSSI prevalence and characteristics, the relationship between NSSI and suicide, the treatments of NSSI. | N/A | -NSSI assessment should be standard practice in medical settings.  -Randomized control trials of effective treatment and prevention strategies are warranted.  -The questions most pressing for clinicians and allied medical health professionals include (a) discerning individuals with NSSI history at elevated risk for suicide from those not at elevated risk, (b) effective treatment regimes, (c) effective prevention strategies in school and community settings, and (d) assessment and referral protocols likely to result in effective treatment and abatement of NSSI behavior. |
| Whitlock, J., & Knox, K. L. (2007).  The relationship between self-injurious behavior and suicide in a young adult population.  *Archives of Pediatrics & Adolescent Medicine*, *161*(7), 634‑640 | Original retrospective study | USA | 2875 college-age students. | To test the hypothesis that self-injurious behavior (SIB) signals an attempt to cope with psychological distress that may co-occur or lead to suicidal behaviors in individuals experiencing more duress than they can effectively mitigate. | Self-report questionnaires:  -K-6 Scale  -Attraction to Life Scale  -Lifetime Prevalence of Suicidality. | -One quarter of the sample reported SIB, suicidality, or both; 40.3% of those reporting SIB also report suicidality.  -Compared with respondents reporting only suicidality, those also reporting SIB were more likely to report suicide, plan, gesture, and attempt.  -Lifetime SIB frequency exhibits a curvilinear relationship to suicidality. |
| Whitlock, J., Muehlenkamp, J., Eckenrode, J., Purington, A., Baral Abrams, G., Barreira, P., & Kress, V. (2013).  Nonsuicidal self-injury as a gateway to suicide in young adults.  *The Journal of Adolescent Health: Official Publication of the Society for Adolescent Medicine*, *52*(4), 486‑492 | Original longitudinal study | USA | 1466 students at five U.S. colleges evaluated annually while three years. | -Analyses tested the hypotheses that the practice of NSSI prior to STB and suicide behavior (excluding ideation) reduced inhibition to later STB independent of shared risk factors.  -Analyses also examined factors that predicted subsequent STB among individuals with NSSI history. | Self-report questionnaires:  -Non-Suicidal Self-Injury Assessment Tool (NSSI-AT)  -Revised Life Orientation Test (LOT-R)  -Emotion Regulation Scale  -Meaning in Life Questionnaire  -Modified Version of the Life History Calendar  -K-6 Scale  Reports of suicidal ideation, behaviors and attempts: N/A. | -History of NSSI did significantly predict concurrent or later STB independent of covariates common to both.  -Among those with prior or concurrent NSSI, risk of STB is predicted by > 20 lifetime NSSI incidents and history of mental health treatment.  -NSSI prior to suicide behavior serves as a "gateway" behavior for suicide and may reduce inhibition through habituation to self-injury. |
| Wilkinson, P. (2013).  Non-suicidal self-injury. *European Child & Adolescent Psychiatry*, *22 Suppl 1*, S75‑79 | Review | England | N/A | This review will first discuss whether it is appropriate to include NSSI as a separate  DSM-5 psychiatric diagnosis. A discussion will follow whether it is appropriate to separate ‘suicidal’ from ‘nonsuicidal self-injury’. | N/A | -The lack of nosological recognition coupled with clear  psychopathological importance is to be recognised in the  5th edition of the DSM.  -We agree that this is appropriate and is likely to have several positive consequences including improving communication between professionals and patients, informing treatment and management decisions and increasing research into the nature, course and  outcome of NSSI.  -We agree that while suicidal  and non-suicidal self-harm are often seen together, they are not the same behaviour and that it is both valid and useful to separate them. |
| Wilkinson, P., Kelvin, R., Roberts, C., Dubicka, B., & Goodyer, I. (2011).  Clinical and psychosocial predictors of suicide attempts and nonsuicidal self-injury in the Adolescent Depression Antidepressants and Psychotherapy Trial (ADAPT).  *The American Journal of Psychiatry*, *168*(5), 495‑501 | Original longitudinal study | England | 164 **adolescents** with major depressive disorder taking part in the **Adolescent Depression Antidepressants** and **Psychotherapy Trial (ADAPT**). | The authors assessed whether **clinical** and **psychosocial** factors in depressed **adolescents** at baseline predict **suicide attempts** and **nonsuicidal** self-injury over 28 weeks of follow-up. | Questionnaires:  -Schedule for Affective Disorders and Schizophrenia for School-Age Children – Present and Lifetime Version (K-SADS-PL)  -Schedule for Affective Disorders and Schizophrenia for Adolescents – Lifetime Version  -Children’s Depression Rating Scale – Revised (CDRS)  Self-report questionnaires:  -McMaster Family Assessment Device – 12 – Item General Functioning Scale  -Cambridge Friendships Questionnaire. | - **Nonsuicidal self**-**injury** over the follow-up period was independently predicted by **nonsuicidal self**-**injury**, hopelessness, anxiety disorder, and being younger and female at entry.  -Both suicidal and **nonsuicidal self**-harm persisted in depressed **adolescents** receiving treatment in the **ADAPT** study.  -A history of **nonsuicidal self**-**injury** prior to treatment is a **clinical** marker for subsequent **suicide attempts** and should be as carefully assessed in depressed youths as current suicidal intent and behavior. |
| Ystgaard, M., Arensman, E., Hawton, K., Madge, N., van Heeringen, K., Hewitt, A., and al. (2009).  Deliberate self-harm in adolescents: comparison between those who receive help following self-harm and those who do not.  *Journal of Adolescence*, *32*(4), 875‑891 | Original retrospective study | Australia, Belgium, England, Hungary, Ireland, The Netherlands, and Norway | 30,532 pupils aged 14-17. | This international comparative study addresses differences between adolescents who engage in deliberate self-harm (DSH) and who receive help following the DSH episode versus those who do not. | Self-report questionnaires:  -Lifestyle and Coping Questionnaire  -Self-Concept Scale  -Plutchik Impulsivity Scale  -Hospital Anxiety and Depression Scale (HADS). | -An act of DSH in the year prior to the study was reported by 1660 participants. Nearly half (48.4%) had not received any help following DSH, 32.8% had received help from their social network only and 18.8% from health services.  -Adolescents who had been in contact with health services following DSH reported more often a wish to die, lethal methods, alcohol/drug problems and DSH in the family compared to those who had not. |
